# Biodegradation of plastics and ecotoxicity testing: when should it be done

**DOI:** 10.3389/fmicb.2014.00475

**Published:** 2014-09-05

**Authors:** Francesco Degli-Innocenti

**Affiliations:** Ecology of Products and Environmental Communication, Novamont S.p.A.Novara, Italy

**Keywords:** biodegradation, environmental, biodegradable materials, ecotoxicology, ecotoxicity, degradation mechanisms, bioplastics, biodegradable plastics

Most biodegradable plastics are water-insoluble solid materials under normal environmental conditions (Bohlmann, 2005). The biodegradation of plastics is a surface erosion process which happens at the solid/liquid interface, where extracellular enzymes, in the liquid phase, start depolymerisation of the solid phase (Tosin et al., 1996). The depolymerisation releases monomers that are assimilated by the surrounding microorganisms (the “*central dogma*” for biodegradation of polymers; Kaplan et al., 1993). The depolymerisation is the limiting factor, while the subsequent assimilation of monomers by the microbes is expected to be immediate (Saponaro et al., 2008). For example, a cube shaped plastic item made with an isotropic and homogeneous biodegradable polymer (a “*Polyester X*”) with a volume of 1 cm^3^ and a density of 1 g/cm^3^ is subjected to biodegradation. When biodegradation starts, the cube will shrink as a consequence of the erosion of the six surfaces. Therefore, the first phenomenon of biodegradation is surface erosion that can be measured as a decrease in thickness per unit of time per unit area (cm day^−1^ cm^−2^). In this simple model, suppose a constant erosion rate of 0.0095 cm day^−1^ cm^−2^. With this erosion rate it takes about 100 days to have a complete biodegradation of the plastic cube. After 16 days, each side of the cube will be reduced to 0.85 cm (Figure 1), the residual volume will be 0.61 cm^3^ (equal to 0.61 g) and 0.39 g of plastic will have being assimilated (i.e., the percentage of biodegradation will be 39%).

**Figure 1:**
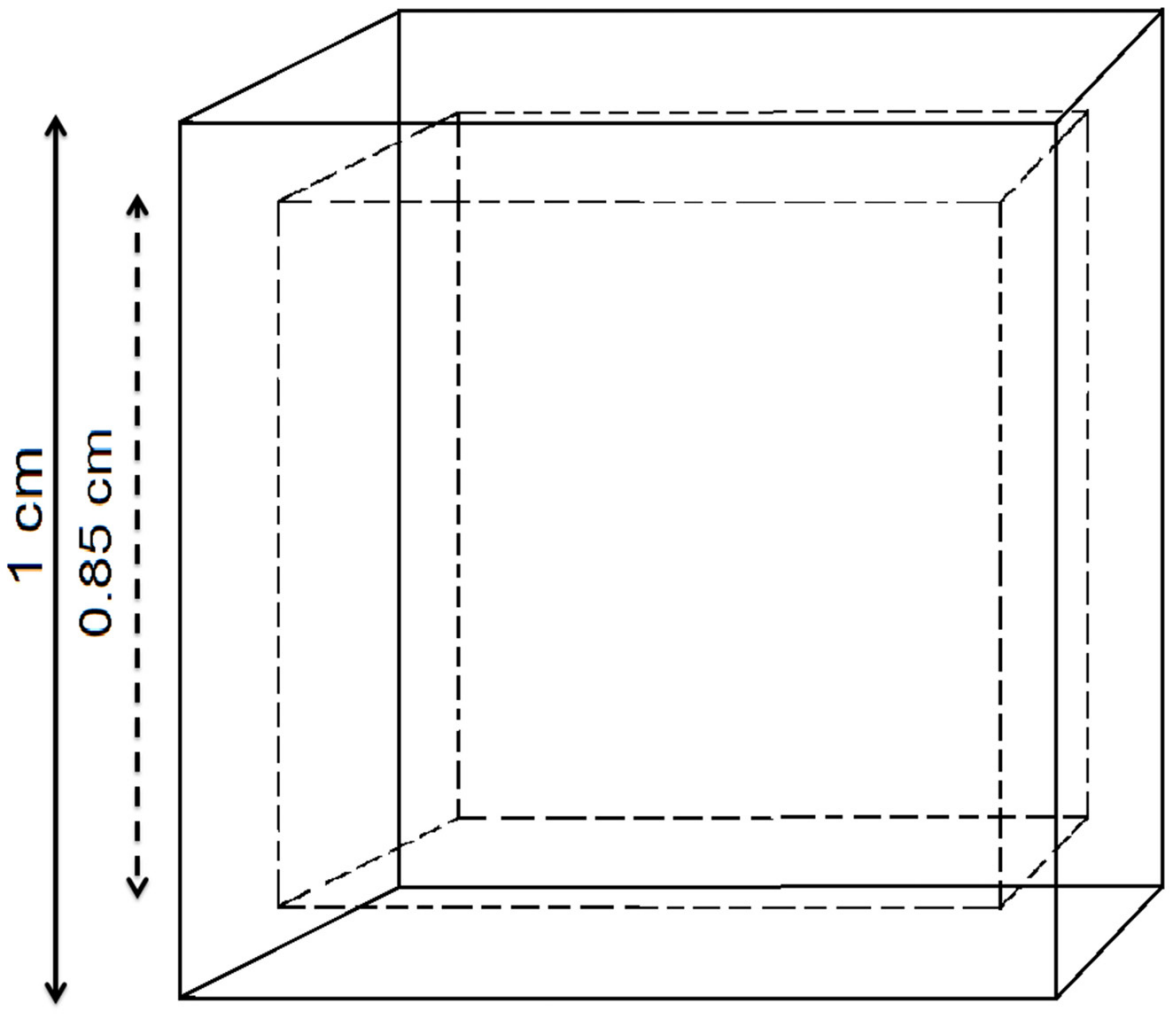
**Biodegradation “shrinks” the plastic cube by means of surface erosion**. The missing part has been totally biodegraded (*B* = 100%), while the residual polymer in the smaller cube is still intact (*B* = 0%).

Assume that the process is stopped at this point. The cube is still a plastic item, intact, apart from the surface, a thin, partly degraded layer where the degradation processes is “frozen” in an intermediate phase. A reduction in molecular weight and a loss of material properties has happened (especially in the amorphous regions, more sensitive to degradation) but without weight loss (Eldsäter et al., 2000) The missing mass has been converted into CO_2_ and H_2_O (for simplicity, we consider that all the plastic is totally mineralised, with no conversion into biomass and no recalcitrant bio-products being formed). Is a percentage of 39% a substantial biodegradation level? If referred to the *Polyester X* 39% sounds incomplete. But if we consider more carefully the matter, it is the cube that it is still to reach a complete biodegradation. Many polymeric chains of the *Polyester X* have been already fully biodegraded. Therefore, the biodegradation percentage of a solid polymer does not provide us with relevant information on the mass that has been depolymerised rather it is just a ratio between the mass that has been metabolized and the original mass. More accurately the biodegradation of the residual cube was 0%, while biodegradation of the missing part was 100%.

In order to understand what happened at the cube at molecular level in the first 16 days of biodegradation we must consider the following reasoning:

Assume the *Polyester X*, which the cube is made of, has an average molecular weight (Mn) of 50,000. The moles of polymeric chains present in 0.39 g are: 0.39 g/(50,000 g/mol) = 7.8 × 10^−6^ mol. This means that: 7.8 × 10^−6^ × 6.022 × 10^23^ = 4.70 × 10^18^ macromolecules have been biodegraded. Still, 7.35 × 10^18^ macromolecules are intact, in the cube. With an erosion rate of 0.0095 cm day^−1^ cm^−2^, a total of 1.14 × 10^17^ molecules are eroded and biodegraded from each cm^2^, every day.

We will now approach the core argument of this paper: ecotoxicity assessment of biodegradable plastics. The established approach is the following. A plastic sample is made to biodegrade in soil under controlled conditions up to a given extent. Then, a sample of soil is collected and tested with a proper array of ecotoxicity tests. In parallel, a sample of soil that has not been exposed to the plastic is tested with the same approach, as a control. The plastic is assumed to be safe for the environment if no difference between the soils are detected. The higher the sensitivity of the exotoxicity tests, the higher the concentration of the potential ecotoxic chemicals, the higher will be the robustness of this assumption. Therefore, the biodegradation test must be set up with the purpose to maximize the release of the potential toxic chemicals in the soil sample. The chance of detecting potential ecotoxic chemicals, depends on the *amount* of polymer that is biodegraded. This is not just dictated by the biodegradation level, since this is a ratio. The number of macromolecules metabolized after 16 days, when the 1 g cube has reached a nominal biodegradation level of 39%, is 390 times higher than the number of macromolecules metabolized from a 1 mg totally biodegraded (100%) sample in 100 days. Therefore, it is clear that neither the test duration nor the biodegradation level are relevant, only the metabolized mass.

The consequence of this is that the duration of the test procedure used to prepare the soil for subsequent ecotoxicity testing can be reduced (with obvious practical advantages) on the condition that the original mass of plastic and the biodegradation rate are both high. A high mass of plastic provides a high amount of macromolecules available for biodegradation (and a higher amount of potential by-products, if any). The biodegradation rate is controlled by the available surface which, in turn, is controlled by the granulometry of the plastics. Thus, plastics must be milled before testing. Best results are obtained by converting the plastics into very thin films, and then fragmenting the films via cryogenic milling.

This approach is applicable when: the full biodegradation of the test material has been already proven; the material is isotropic. A material that is made in layers will not behave as the homogeneous “cube” shown in our example. All the layers must be subjected to biodegradation in this case. A material whose biodegradability is not known must be totally degraded before applying the ecotoxicity testing.

The necessity of testing high concentrations of test material can in turn be problematic. The sudden addition of high amounts of biodegradable materials represents a disturbance to the soil environment. It is important to note that fresh organic matter (any organic matter, not just biodegradable plastics) will cause transient “toxic” effects in soil if added in high amounts (Zucconi and de Bertoldi, 1987). Starch, straw, bio-waste, etc. can be “ecotoxic” if tests are carried out during the biodegradation phase. It is known that natural substances, such as polysaccharides, etc., can cause transient phyto-toxicity during degradation due to the fact that their degradation can cause oxygen depletion in soil (negative for roots) and the production of metabolic intermediates. This can create short-term unfavorable conditions to soil organisms and plants. Thus, there is a risk to get false positives (“positive” in this context means to pinpoint an ecotoxicity effect) caused by the excessive biodegradation triggered by the unnatural addition of huge amount of biodegradable material in the soil. To make any comparison more balanced, the reference must be a soil treated in a similar way. This can be achieved by adding to the soil a GRAS (Generally Recognized As Safe) biodegradable material at the same concentration as the test plastic material. Microcrystalline cellulose is the natural candidate for this purpose because it is biodegradable, safe, solid, and already used as a reference in biodegradation tests.

To conclude: (i) the ecotoxicity testing should be carried out on samples of soil where the test plastic material has been added in high concentration (to get a high amount of potential ecotoxic by-products, produced during biodegradation); (ii) the plastic material should be milled in order to get a high surface and a higher biodegradation rate (to reduce testing duration); (iii) a soil where a GRAS material (i.e., cellulose) has been biodegraded should be used as a control, to take into account the effects due to soil disturbance, that could otherwise be mistaken as toxic effects caused by the plastic.

## Conflict of interest statement

The author declares that the research was conducted in the absence of any commercial or financial relationships that could be construed as a potential conflict of interest.

## References

[B1] Bohlmann G. M. (2005). General characteristics, processability, industrial applications and marke evolution of biodegradable polymers, in Handbook of Biodegradable Polymers, ed Bastioli C. (Shrewsbury; Shropshire: Rapra Technology Limited Shawbury), 183

[B2] Eldsäter C. Erlandsson B. Renstad R. Albertsson A.-C. Karlsson S. (2000). The biodegradation of amorphous and crystalline regions in film-blown poly(ϵ-caprolactone) Polymer 41, 1297–1304 10.1016/S0032-3861(99)00278-522806733

[B3] Kaplan D. J. Mayer J. M. Ball D. Mcmassie J. Allen A. L. Stenhouse P. (1993). Fundamentals of biodegradable polymers, in Biodegradable Polymers and Packaging, eds Ching C. Kaplan D. L. Thomas E. L. (Basel: Technomic Publication), 1–42

[B4] Saponaro S. Sezenna E. Degli Innocenti F. Mezzanotte V. Bonomo L. (2008). A screening model for fate and transport of biodegradable polyesters in soil. J. Environ. Manage. 88, 1078–1087 10.1016/j.jenvman.2007.05.010 17624656

[B5] Tosin M. Degli Innocenti F. Bastioli C. (1996). Effect of the composting substrate on biodegradation of solid materials under controlled composting conditions. J. Environ. Polym. Degr. 4, 55–63 10.1007/BF02083883

[B6] Zucconi F. de Bertoldi M. (1987). Compost specifications for the production and characterization of compost from municipal solid waste, in Compost: Production, Quality and Use, eds De Bertoldi M. Ferranti M. P. L'Hermite P. Zucconi F. (London; New York, NY: Elsevier Applied Science), 30

